# Surgical Treatment for a Full-Thickness Macular Hole That Developed on a Large Drusenoid Pigment Epithelial Detachment

**DOI:** 10.7759/cureus.15785

**Published:** 2021-06-20

**Authors:** Kunihiro Azuma, Tomoyasu Shiraya, Fumiyuki Araki, Satoshi Kato, Shigeko Yashiro, Miyuki Nagahara, Takashi Ueta

**Affiliations:** 1 Ophthalmology, Graduate School of Medicine and Faculty of Medicine, The University of Tokyo, Tokyo, JPN; 2 Ophthalmology, National Center for Global Health and Medicine, Tokyo, JPN

**Keywords:** drusenoid pigment epithelial detachment, inner limiting membrane, macular hole, vitrectomy, age-related macular degeneration

## Abstract

Full-thickness macular hole (FTMH) and age-related macular degeneration (AMD) can affect the same eyes in the older population. Previously reported phenotypes of AMD concurrent with FTMH include early/intermediate AMD and serous pigment epithelial detachment (PED). A 68-year-old woman presented to our clinic with decreased vision due to a cataract and a large drusenoid PED in both eyes. After ruling out choroidal neovascularization, she underwent cataract surgery. Three days after the cataract surgery, an FTMH was found in the left eye. Although the FTMH was not closed after the initial pars plana vitrectomy (PPV) with the inner limiting membrane (ILM) peeling and air tamponade, it was closed after reoperation with additional ILM peeling, retinal massage, and SF6 gas tamponade. Best-corrected visual acuity (BCVA) was improved from 20/60 before the first PPV to 20/40 at six months after the reoperation. Some large soft drusen in the macula were fused after surgeries in the operated eye, but not in the fellow eye. An FTMH concurrent with a large drusenoid PED is rare. It can be closed surgically, and postoperative visual function can improve.

## Introduction

Drusenoid pigment epithelial detachment (PED) is one of the characteristic features of age-related macular degeneration (AMD). Drusenoid PEDs are formed by the confluence of large drusen, as distinguished from other subtypes of PED, including serous and vascularized PED, and are associated with a higher risk of progression to late AMD [1]. According to age-related eye disease study 2 (AREDS2), the prevalence of drusenoid PED among patients with the category-three intermediate AMD is considered to be less than 3% [1].

A full-thickness macular hole (FTMH) is a common macular pathology found among those aged >40 years, and it is typically caused by posterior hyaloid contraction, perifoveal vitreous detachment, and anteroposterior vitreoretinal traction [2]. Pars plana vitrectomy (PPV) with the inner limiting membrane (ILM) peeling and air/gas tamponade has been established as an effective standard treatment [3].

Here we describe an unusual case of an FTMH that developed on a large drusenoid PED with multimodal imaging. The unusual FTMH was successfully closed after surgeries.

## Case presentation

**Figure 1:**
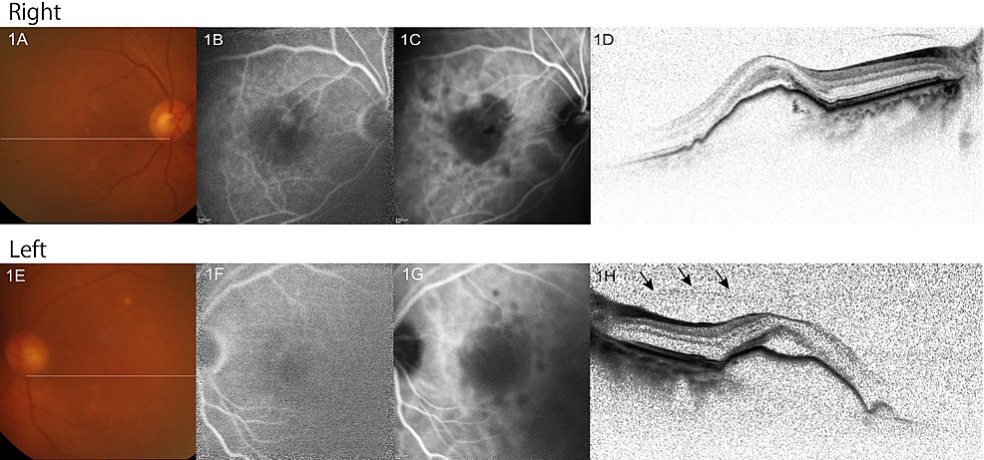
Color fundus photo (1A,1E), fluorescein and indocyanine green angiography at 6-7 min (1B,1C,1F,1G), and OCT (1D,1H) at initial presentation. Although cataracts limited transparency, drusenoid pigment epithelial detachment without neovascularization was observed in both eyes. In addition, vitreous adhesion at the fovea and optic nerve head was shown on OCT (indicated by arrows for the left eye in 1H). OCT, optical coherence tomography

A 68-year-old woman was referred to our ophthalmic clinic due to gradual deterioration in visual acuity. Her best-corrected visual acuity (BCVA) was 20/200 in both eyes. Ophthalmic evaluations revealed advanced cataracts and large drusenoid PEDs with shallow serous retinal detachment in both eyes. Fluorescein and indocyanine green angiography did not show retinal or choroidal neovascularization (Figure 1).

**Figure 2:**
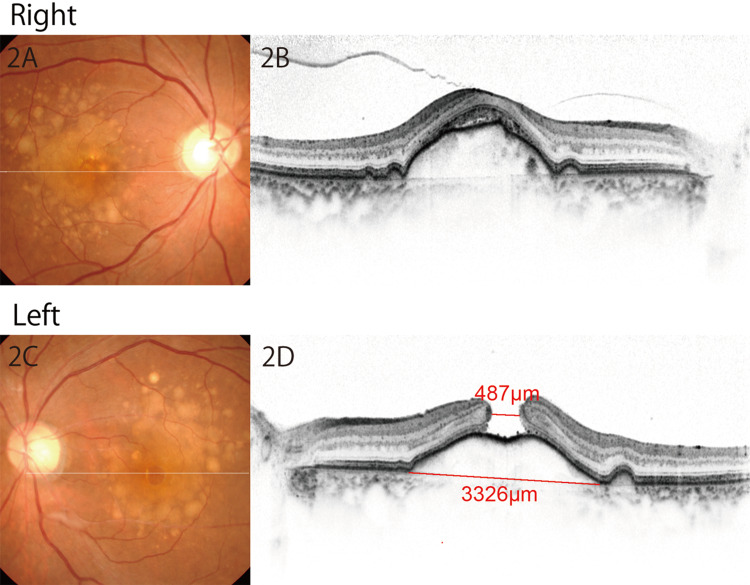
Color fundus photo (2A,2C) and OCT (2B,2D) after cataract surgery in both eyes. An FTMH was identified in the left eye. FTMH, full-thickness macular hole OCT, optical coherence tomography

The axial length was 22.53 mm in the right eye and 22.33 mm in the left eye. She was a current smoker and did not have any other systemic comorbidity. In both eyes, the vitreous adhered to the fovea and optic nerve on optical coherence tomography (OCT) (Figure 1). Phacoemulsification and intraocular lens implantation were performed in both eyes with no intrasurgical complication. Three days after the surgery, BCVA was improved to 20/22 (0.9 by decimal) in the right eye; however, BCVA was 20/60 (0.3 by decimal) in the left eye, and OCT conducted on the same day revealed a stage-four FTMH (minimum diameter 480 µm) developed on a large drusenoid PED (minimum diameter 3300 µm) (Figure 2).

**Figure 3:**
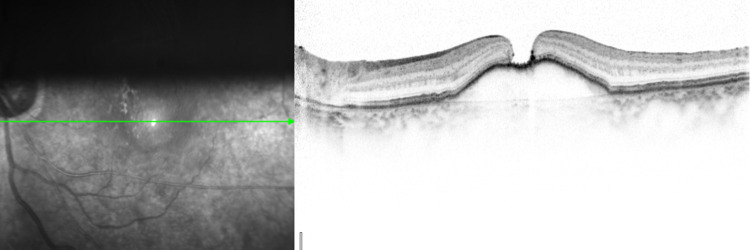
A horizontal section of OCT after initial PPV in the left eye. An FTMH remained open. OCT, optical coherence tomography; PPV, pars plana vitrectomy; FTMH, full-thickness macular hole

 The PPV with Brilliant blue G-assisted ILM peeling and air tamponade was performed. However, because the macular hole (MH) was not closed (Figure 3), reoperation was performed two weeks later.

**Figure 4:**
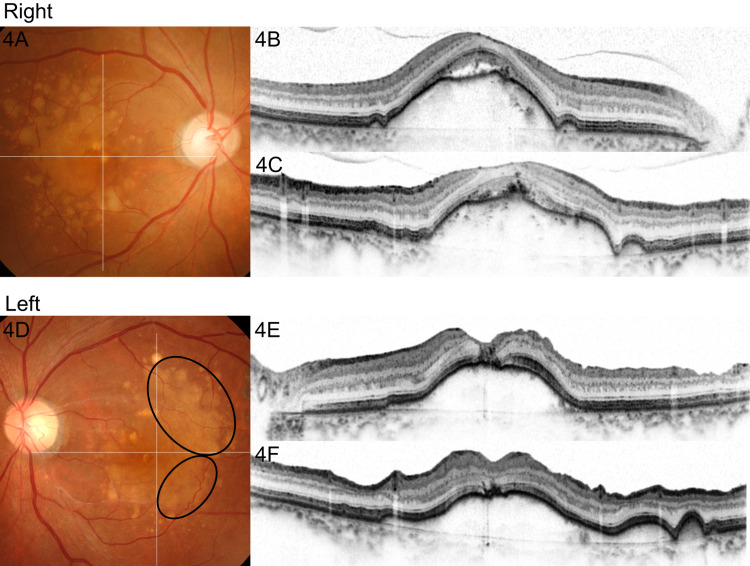
Color fundus photo (4A,4D) and OCT of horizontal (4B,4E) and vertical (4C,4F) sections after surgeries. An FTMH on a drusenoid PED in the left eye was closed after the second PPV in the left eye (4D,4E,4F). Large soft drusen in the temporal macula in the left eye were fused with each other (circles, 4D vs. 2C). On the other hand, the distribution of drusen did not change in the right eye during the same period of follow-up (4A vs. 2A). OCT, optical coherence tomography; PED, pigment epithelial detachment; PPV, pars plana vitrectomy; FTMH, full-thickness macular hole

Additional ILM peeling in the temporal macula, retinal massage around the fovea using a back-flush needle to reduce the hole size, and SF6 gas tamponade were performed. After the reoperation, the FTMH was closed and BCVA was modestly improved to 20/40 at six months after the reoperation (Figure 4). Interestingly, a comparison of color fundus photographs taken before the first PPV (Figure 2C) and after the second PPV (Figure 4D) revealed that large soft drusen in the temporal macula of the left eye were fused with each other after surgeries. In contrast, soft drusen in the right eye were stable during the same period of time (Figure 4A vs. Figure 2A).

## Discussion

Several studies have reported the surgical outcomes of FTMHs in eyes with early and intermediate drusen [4-6]. However, therapeutic outcomes of FTMHs developed on a large drusenoid PED have not been reported to date. Berinstein et al. reported the surgical outcomes of 34 cases in which an FTMH developed in eyes with drusen that were categorized as either early (category two) or intermediate (category three) AMD according to AREDS [4]. In their study, the closure rate after PPV without ILM peeling was 76%, which was lower than in their idiopathic case series (89%), and the MH closure rate became lower depending on the extent of the drusen. Recently, Michalewska and Nawrocki compared the outcomes of inverted flap technique in MH surgery among 18 eyes with intermediate AMD (defined as at least one large drusen, 20 medium-sized drusen, or 65 small drusen) and those without AMD [5]. The closure rate in eyes with intermediate AMD was 88%, which was lower than for idiopathic MH (98%). The lower chance of MH closure in eyes with drusen might have been in line with the current case that needed reoperation to achieve MH closure. Another recent study reported surgical outcomes of 27 MH cases in the eyes with early or intermediate AMD [6]. The study reported a 96.3% closure rate after initial surgery using conventional ILM peeling and gas tamponade, while there were two cases of AMD progression.

On the other hand, Panthier et al. reported a peculiar case of a combined FTMH and retinal pigment epithelial (RPE) tear beneath the hole in an eye with drusenoid PED, in which no therapeutic intervention was conducted due to the patient’s refusal of treatment [7].

Several cases of an FTMH developing on a serous PED have been reported. Such FTMHs sometimes developed following intravitreal injections of anti-vascular endothelial growth factor (VEGF) antibodies [8-9]. In the case where Raiji et al. reported, pre-existing vitreomacular traction before the development of an FTMH was shown in OCT images [8]. In some of the operated eyes, FTMHs were closed and visual acuity was modestly improved [8-10]. In the other eye, the hole was not successfully closed after surgery [10].

Regarding the pathogenesis of FTMH development in eyes with drusenoid PED, a previous study suggested that different mechanical forces were involved in the pathology: in addition to anteroposterior/tangential force at the vitreomacular interface, a retracting force within PED due to absorption/flattening of drusenoid PED, or a force pushing out of the drusenoid PED as it grew, might have played a role [7]. In our patient, because the size or height of the drusenoid PED did not change before PPV, we speculated that forces at the vitreomacular interface could be causative for the FTMH that developed on the drusenoid PED. Indeed, vitreous adhesion at the fovea was shown on OCT at the initial presentation. Cataract surgery might have had some effect on the vitreoretinal interface and may have facilitated FTMH development, although we could not confirm whether the FTMH developed just before or after the cataract surgery.

We needed reoperation to close the MH in our patient. In line with this, previous studies have indicated a trend toward lower MH closure rates in eyes with drusen [4-5, 10]. The degenerative RPE associated with AMD might play a role in preventing normal MH closure which is usually seen in idiopathic MH. A previous study discussed that the defective RPE might be unable to drain the subretinal fluid after surgery, or the damaged RPE might hinder the production of healing mediators [4]. Or we speculate that convex curvature due to PED rather than normal concave curvature of the fundus may make MH closure even more difficult. Taken together, measures including inverted ILM flap technique and gas tamponade rather than air might have been useful to achieve successful MH closure after initial PPV.

## Conclusions

A rare case of FTMH that developed on a large drusenoid PED was described, for which surgical treatment was effective for MH closure and visual acuity (VA) improvement. Since the closure rate might be lower in AMD-associated FTMHs, precautious measures such as a larger area of ILM peeling, an inverted ILM flap, and a gas tamponade instead of air, should be considered.
